# Supplementary figures and images for: Soft soled footwear has limited impact on toddler gait
Source: PLoS One. 2021 May 10;16(5):e0251175. doi: 10.1371/journal.pone.0251175 (PMC8109762; doi:10.1371/journal.pone.0251175)

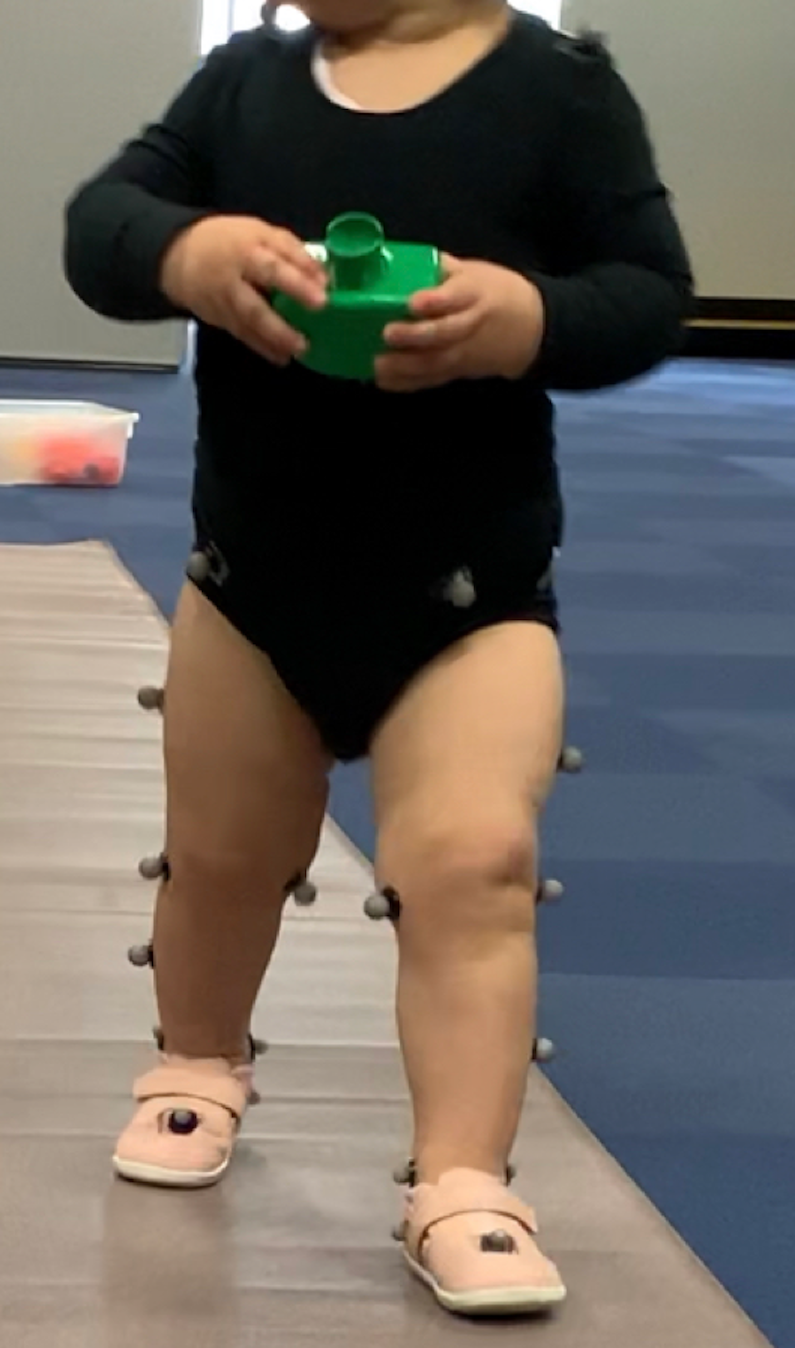

Supplement: S1 Fig — (TIF) [file pone.0251175.s001.tif]

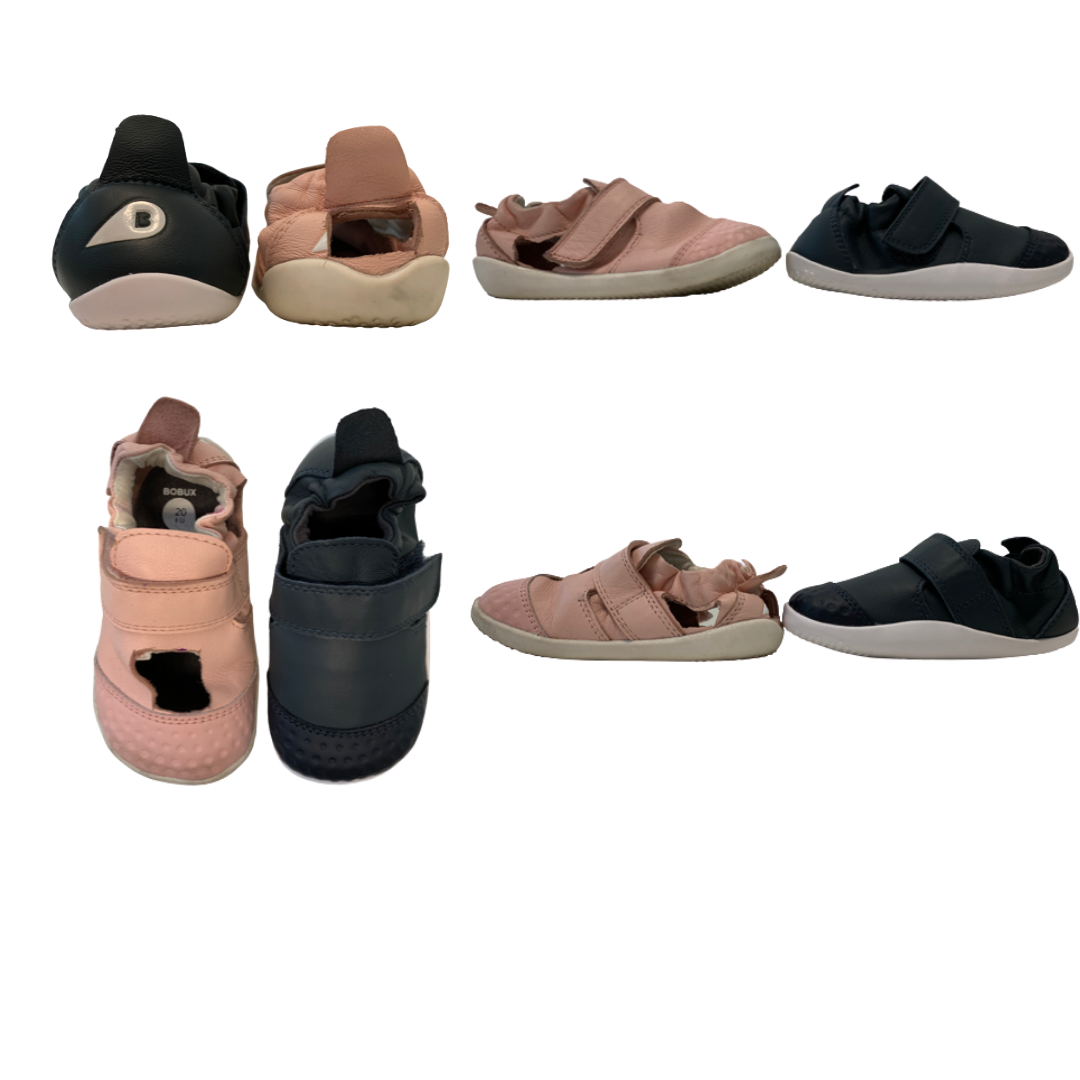

Supplement: S2 Fig — (TIF) [file pone.0251175.s002.tif]
