# Supplementary material for: Soft soled footwear has limited impact on toddler gait
Source: PLoS One. 2021 May 10;16(5):e0251175. doi: 10.1371/journal.pone.0251175 (PMC8109762; doi:10.1371/journal.pone.0251175)
Supplement: S1 File — (DOCX) [file pone.0251175.s003.docx]

**S1 File.** **Gait variables and their description**

| Spatial Measures | Variable Description |
| --- | --- |
| Stride length (cm) | From the heel points of two consecutive steps of the same foot |
| Toe in/toe out (°) | The angle between the middle of the steps and the middle of the foot |
| Temporal Measures |  |
| Stride time (sec) | Time between the first contact of one foot to the first contact of that foot again during walking |
| Step time (SEC) | Time between the first contact of one foot to the first contact of the other foot during walking |
| Velocity (cm/sec) | The distance walked divided by the speed of walking |
| Swing percentage (%) | Percentage of the gait cycle when foot is not in contact with ground |
| Stance percentage (%) | Percentage of the gait cycle with feet in contact with the ground |
| Double support time (sec) | Time during walking when both feet are on the ground |
| Cadence (steps/minute) | Number of steps taken over a minute |
| Steps (COunt) | Number of steps taken over testing session |
| Kinematic Measures |  |
| Peak hip flexion (°) | The maximal flexion angle of hip joint during stance phase of walking |
| Peak hip extension (°) | The maximal extension angle of hip joint during stance phase |
| Total hip flexion/extension rOM*(°) | The difference between peak hip flexion and extension during stance phase |
| Peak hip adduction (°) | The maximal adduction (towards the midline) angle of the hip joint during stance phase |
| Peak hip abduction (°) | The maximal abduction (away from the midline) angle of the hip joint during stance phase |
| Total hip adduction/ abduction ROM* (°) | The difference between peak hip adduction and abduction during stance phase |
| Peak hip internal rotation (°) | The maximal internal rotation angle of the hip joint during stance phase |
| Peak hip external rotation (°) | The maximal external rotation angle of the hip joint during stance phase |
| Total hip internal/external rOM (°) | The difference between peak hip internal and external rotation during stance phase |
| Peak knee flexion (°) | The maximal flexion angle of the knee joint during stance phase |
| Peak knee extension (°) | The maximal extension angle of the knee joint during stance phase |
| Total knee flexion/extension ROM (°) | The difference between peak knee flexion and extension during stance phase |
| Peak ankle flexion (°) | The maximal flexion angle of the ankle joint during stance phase |
| Peak ankle extension (°) | The maximal extension angle of the ankle joint during stance phase |
| Total ankle flexion/ extension ROM (°) | The difference between peak ankle flexion and extension during stance phase |
| Peak subtalar inversion (°) | The maximal inversion angle of the subtalar joint during stance phase |
| Peak subtalar eversion (°) | Maximal eversion angle of the subtalar joint during stance phase |
| Total subtalar inversion/ eversion ROM(°) | Difference between peak subtalar inversion and eversion during stance phase |

*Range of Motion
