# Supplementary material for: Soft soled footwear has limited impact on toddler gait
Source: PLoS One. 2021 May 10;16(5):e0251175. doi: 10.1371/journal.pone.0251175 (PMC8109762; doi:10.1371/journal.pone.0251175)
Supplement: Attachment — Submitted filename: Toddler PLOS Review R1.docx [file pone.0251175.s005.docx]

4^th^ January 2021

Riccardo Di Giminiani

Academic Editor, PLOS One

Dear A/Prof Di Giminiani,

Re: Manuscript ID: PONE-D-20-25753

We thank you and the reviewers for their comments and for the opportunity to revise our manuscript. What follows is a point by point response to each comment. Where we have disagreed with the reviewer’s comments we have provided a detailed rebuttal justifying our decision.

**Editorial comments:**

We have revised the format of the article as per the style requirements.

We have provided captions for the Support Information file at the end of the manuscript and updated the intext-citation of this accordingly.

We note PLOS-Ones preference for protocols and have added and referenced this within the manuscript.

We have responded to the reviewers and referenced the line numbers where changes were made.

We provided an updated financial disclosure section and competing interests statement confirming we adhere to all PLOS ONE policies on sharing data and materials, including the required statement as below:

**Updated competing interest statement:**

This research was partially supported by from Bobux Pty Ltd, and this funding was administered by Monash University. CMW has received prior research funding from Bobux Pty Ltd, administered by Monash University, outside this submitted work. All other researchers declare no competing interests. This competing interest statement does not alter our adherence to PLOS ONE policies on sharing data and materials.

**Reviewer #1: 1. Introduction:**

**a. For children, the selection of footwear ranges from very soft to very stiff. But what about the options for infants? Is there very stiff footwear for infants on the market? Is that popular and beneficial to infants?**

Response: There are variable footwear types commercially available for young children. We have detailed within the methods that the size of commercially available footwear tested was a EU Sz 20. At the time of this response, we are able to find within common online footwear stores a variety of footwear in this size ranging from sneakers, boots, joggers, gumboots, sandals and similar styles shoes to those tested within this research. We have included a statement at Page 3, Lns 78-80, highlighting the variety available to parents:

*There are numerous types of footwear commercially available in toddler foot sizes, including boots that cover the ankles with firm soles, sandals with variable sole flexibility and limited upper coverage, or pre-walkers styles with covered uppers and flexible soles.*

**b. The authors cited two studies from a same toddler study which compared the softest footwear and the stiffest footwear. How were these two footwears different from a mechanical test?**

Response:

Additional information is provided about the footwear within these two studies on Pages 4-5, Lns 102-114. We have added:

*Footwear of interest was categorised by its flexibility relating to the amount of degrees per newtons were required to twist the footwear to 45^o,^ and tested with a custom built testing jig. Footwear with the highest torsional flexibility (~70^o^/Nm) (14) resulted in a shorter stance time (13), wider step width (13) and higher peak plantar pressures (14) in compared to the most footwear with the stiffer response to torsional testing (~30^o^/Nm).*

**c. The author agreed that different shoe soles can lead to different walking patterns in children. However, there is no rationale on why soft shoe sholes were selected for this study.**

**Response:**

We have included the statement on Page 5 Ln 128:

*This footwear type was chosen given there is no research evaluating its impact on gait, despite widespread use.*

**d. Review of gait development in newly walking infants is not complete.**

**Response:**

Although we are unsure of which aspects of gait development the reviewer is alluding to, we have made a number of amendments to our first paragraph to provide a greater description of the development of gait in newly walking infants. We hope that the reviewer finds this satisfactory.

We have provided details on age of gait acquisition, velocity, cadence, arm swing through transitory movements and coordination patterns. We have also added discussion about the joint angles with the following statements on Page 3 Lns 61-65 and Lns 68-70:

*There is a transition period between crawling and walking where independent walking is refined. During this time, toddler’s spend time perfecting standing, side stepping and may practice walking holding a trolley or a parent’s hand. It is not until toddlers are walking without this support, are they considered independent walkers (1).*

AND

*Toddlers with an immature gait pattern also commonly walk with greater knee flexion and greater ankle flexion during loading (2), and this matures to an adult pattern by 2 years of age.*

**e. There is no hypothesis to test in this study.**

Response:

We have included the statement at Page5, Lns 129-131:

*We hypothesized that soft-soled footwear would result in a difference in the common gait variables, similar to the differences seen in older children demonstrating confident walking in footwear compared to walking without footwear.*

2. Methods:

**a. Participants were toddlers with walking experience less than 16 weeks. What was the definition of walking onset? Over the first few months after walking onset, gait pattern changes drastically in newly walking toddlers. So, it may not be appropriate to consider all the subjects in a homogeneous group for data analysis.**

Response:

We have clarified independent walking in the introduction and have included this in the methods section on Page 6, Line 143. We defined walking with the parent’s during recruitment as total walking independence without parental or equipment support on Page 6, Ln 144.

**b. Marker placement: Have this marker placement been used by any previous studies? Cite any references if possible.**

Response: As we have outlined in our methods (Page 7, Lines 166-169), there is no accepted kinematic model for this population. To our knowledge, only one other research team has used a similar marker model in this age group, however they have not published results as yet. To acknowledge this, we have cited their research protocol and provided the following statement:

*The marker placement locations were chosen based on a similar protocol recently published by a research team investigating gait acquisition in young children (17). The marker placement was also similar to the marker position protocols used with older children (18).*

**c. Apparently, there are many shoe brands on the market. Provide a rationale on the selection of these commercially available shoes.**

Response:

We have provided this based on your suggestion both in the introduction as previously requested but also an additional statement (Page 7, Lines 180-182) within the footwear section.

*This shoe was chosen due to its world-wide availability and likeness to other country specific brands, thus improving the generalisability of our findings.*

**d. All the variables in Table 1 are common gait variables. So, this table should be included as a supplemental table in the Appendix.**

Response: We have moved this data to supplementary files based on your suggestion and referenced this at Page 9, Line 228.

**e. Did you run a preliminary analysis between the left and right sides and determine that there was no statistical difference between the two sides in the dependent variables?**

Response: We did as per our protocol and have added this statement at Page 10, Line 225-226.

*While measures were collected for both limbs, there were no statistically significant differences between left and right limb variables.*

**f. Some references are needed to support the rationale on not normalizing the data by height or leg length.**

**Response:**

We have analysed the data in a way that adjusting for variables like the minimal variation in height is unnecessary as we have only ‘tested’ the child against themselves. We have described this within the analysis through the statement regarding clustering by participant within the linear regression model, and have now provided a reference for this type of analysis, and its assumptions (Page 10, Line 251). This method of analysis has been used in other gait papers for within participant child related data with a single time-point/short term intervention including:

- Cranage S, et al. A comparison of young children’s spatiotemporal measures of walking and running in three common types of footwear compared to and bare feet. Gait Posture. 2020; Volume 81, Pages 218-224
- Williams C.M, et al, 2016, Whole body vibration results in short-term improvement in the gait of children with idiopathic toe walking, J Child Neuro, 31 (9), 1143-1149
- Michalitsis J, et al, 2019 Full length foot orthoses have an immediate treatment effect and modify gait of children with idiopathic toe walking, Gait Post 68, 227-231

**g. What was the statistical model? What was the significance level?**

Response:

This has been provided within the manuscript (Page 10, Line 254). We have also added the following sentence:

*Statistical significance was considered as p<0.05*

**3. Results:**

**a. Walking experience 7.2 (SD 2.8) weeks can be a big confounding factor in data interpretation.**

Response:

We agree and have discussed this in great detail as a limitation within the discussion section of the paper (Page 16, Lines 342-345). We have also provided additional suggestions for future research with this age group in how to minimise the variability with toddler gait. As limited research has been done in this age group with this type of gait analysis, we were unsure about the feasibility of gait analysis with this age group and therefore recruited a wider age group. We have added recommendations for future research to cluster participants by time spent, or narrow the time spent walking this now that we know this research is feasible (Page 16, Line 349)

**b. Effect size should be a secondary statistical variable.**

Response:

We agree, and have further highlighted this within the data analysis section. No change has been made in the results. We have included a statement (Page 10, Ln 258) within the data analysis section reading:

*Cohen’s d effect sizes were considered as a secondary statistical variable and categorised as small (d<0.50), medium (d=0.50-0-79), or large (d≥0.8) (25).*

**4. Discussion:**

**a. Velocity is an important gait variable and indicates walking ability. It seems that footwear does not change velocity; rather, it modifies some gait variables.**

Response:

We agree that velocity is an important indicator of walking ability, it has been observed as changing with footwear in older children and adults. Thus, the finding of no difference in early walkers is indeed interesting. We have highlighted this in our opening paragraph in the discussion (Page14, Line 305) as follows:

*“The results showed limited differences in spatiotemporal parameters of gait between conditions, including key variables such as velocity.”*

**Reviewer #2: The authors gather biomechanics data from (adorable!) toddlers with and without shoes, and look for differences in these data. As this area has had little research, the study is exploratory and did not, I believe, have a priori hypotheses. The no doubt quite difficult to carry out study is well designed and the topic is novel.**

Response:

We thank the reviewer for their encouraging kind words. We are in total agreement that this project recruited adorable toddlers. It was indeed a very challenging protocol and we have now included the protocol reference approved by our ethics committee (Page 5, Line 134) and described where it deviated (Page 11, Line 282), however one we refined and are confident could be replicated based on how we have described it and it’s modifications with this paper. Please note, in response to feedback from Reviewer #1 we have now included our original broad hypothesis.

**The work is publishable with one potentially major issue and the rest minor.**

**The only major issue is whether a Bonferroni or other multiple comparisons correction should have been applied to the statistics. A lot of tests are made; should their p-values not be corrected? As this is not my primary area, I do not know the answer with certainly - would like to hear the authors' view. Even if this removed the significance of, e.g., the knee flexion, the results are still interesting and should be published, with commentary in discussion as it is speculating about reasons for the observed changes.**

Response:

Thank you for the opportunity to consider this question and rebut why we have not used this correction. There are times that the Bonferroni correction could be considered, and used in gait analysis research. However, it would not be appropriate to use it with this study for a number of reasons based on the design, hypothesis (a knowledge of impact of footwear on older children’s gait) and how we handled the data during analysis.

Firstly, the Bonferroni or similar multiple comparison tests are used to reduce the chance of Type I errors during ANOVA or MANOVA. We analysed this data with linear regression models as discussed in reference Reviewer 1’s question on normalisation. This alternative method of analysis allowed us to minimise the introduction of Type I errors during the analysis itself with the use of a robust variance estimates. The use of the Bonferroni's correction in this case would most likely introduce even more Type II errors (Rothman, 1990).

Secondly, this correction method should only be considered based on the hypothesis (and it’s outcomes) and study design. In reference to our study, we had a pre-planned aim and hypothesis, in particular, that our hypothesis being we would have variable differences in gait outcomes between wearing footwear and not wearing footwear. Bonerroni corrections should be considered when the null hypothesis is that all variables would be, or all not be, different (Perneger, 1998)

References:

Rothman KJ. No adjustments are needed for multiple comparisons. Epidemiology. 1990;1:43–46.

Perneger TV. What's wrong with Bonferroni's adjustment. BMJ 1998; 316: 1236–1238.

**Minor things to consider:**

**p8: are toddler bodies isometrically scaled version of adult bodies? i think they may be slightly different in arm lengths. does this mean the applications of the generic model could affect the results? Presumably if the same model used for all subjects and both conditions, there is less likely a bias.**

Response:

This assumption of arm length difference is correct for toddlers compared to older children and adults. However, please note that we have not used any variable or marker for arm length therefore this is unlikely to affect our results.

Torso/leg length ratio is also a scaling difference between toddlers, older children and adults, but as the reviewer rightly points out, we are not comparing data between different age groups but to similar children of similar size and torso/length ratio. We have discussed this in more detail on page 10, Line 234.

The segment lengths within the generic model was therefore adjusted and customised model to account these differences, as no other models fitting this size have been developed. We would also highlight that we tested this model to minimise the difference between the scaling and measured markers. We have referenced the rational for doing this as part of standard practice where participant anthropometrics do not match the model (Page 10, Line 239). We have not amended the manuscript in response to this and hope this allays the reviewer’s concern.

**p9: does getting the kinematics from fitted models suppress any important varation? I understand individual models is definitely not possible!**

Response: The kinematic model was participant-specific as we scaled the segments length of a generic model to those of each participant using the markers captured during the static trial. We reworded the following statement on Page 11 to better clarify this.

*The segment lengths of a generic model (built-in model ‘Gait2392-Simbody’ of OpenSim software) were scaled to those of the toddlers using the markers captured during the static trial.*

Furthermore, we employed a within-subjects design and analysis clustered by individual participants. These methodological features ensured we captured and retained as much individual variation within our analyses as practicable.

**p9 typo - maker -> marker**

**p15: grammar: "given difference" ->"Given that the observed differences..."**

Response:

These type/grammar errors have been amended.
