# Supplementary material for: Soft soled footwear has limited impact on toddler gait
Source: PLoS One. 2021 May 10;16(5):e0251175. doi: 10.1371/journal.pone.0251175 (PMC8109762; doi:10.1371/journal.pone.0251175)
Supplement: Attachment — Submitted filename: Toddler PLOS Review R2.docx [file pone.0251175.s006.docx]

7^th^ April 2021

Riccardo Di Giminiani

Academic Editor, PLOS One

Dear A/Prof Di Giminiani,

Re: Manuscript ID: PONE-D-20-25753

We thank you and the reviewers for their comments and for the opportunity to revise our manuscript. What follows is a point by point response to each comment for Reviewer 3. We also thank Reviewer 2 for their positive comments.

**Reviewer #3:**

**1. The authors mention kinetic data in the abstract but don’t provide information on how these data were acquired (i.e., they state that they used GAITRite to collect spatiotemporal data and Vicon to collect kinematic data, but not how they acquired kinetic data).**

*Response*

Thank you for finding this within the abstract. We have deleted this comment as it was added in error, and therefore the reason it was not presented within the manuscript.

**- Line 48: remove the apostrophe in toddlers (i.e., it should read toddlers not toddler’s).**

*Response*

Amended

**- Line 107: What do the authors mean by “confident” walking? This is unclear.**
*Response*

On reflection, this term could create confusion. We have removed it as it relates more to older children and their walking

**- Did the authors ask parents whether and how often their toddlers wore their shoes at home? I wonder if some toddlers may have more experience walking in shoes than others.**

*Response*

We did not consider this during the design phase of our research. We would highlight that we did consider home shoe use during the interpretation of our results, and we alluded that familiarity with footwear may have introduced artifacts or variability (please see Lines 363-364). We have also encouraged researchers undertaking research with todders in the future to consider habituation with footwear to minimise the possibility of home shoe use influencing study outcomes (Lines 365-368).

**- Again, in the abstract, there was mention made of kinetic data, but this is not in the method section.**

*Response*

As previously mentioned, we did not collect this data. Its inclusion in the abstract was an error and has now been removed.

**- Please clarify the clothing on which markers were placed (line 176). Were all toddlers wearing a onsie? I ask because markers placed directly onto clothing can move easily and possibly not reflect true body movement.**

*Response*

Thank you for the opportunity to clarify this. There were no markers on clothing on the lower limb, only the shoulders (for some children who did not want their singlet removed) and nappy area. We have added the following at Lines 184-188 to better explain this:

*We preferred toddlers wear only a nappy for testing, however due to variable laboratory temperatures our of our control, some toddlers wore a singlet or upper body covering along with their nappy. All lower limb markers were on bare legs. The reflective markers were adhered to the skin (or shoulder fabric/nappy) over the semi-permanent mark using double sided tape.*

**- Please explain why stride length was used instead of step length. Literature shows that newly walking toddlers demonstrate variability from step to step suggesting that step length is a better measure to use.**
*Response*

We used stride length to facilitate comparison with previously published data. On reflection, we now have provided right limb step length and time within Table 1, and amended the results section in lines 288-290: We have also provided additional information to understand any variability in response to the comment below.

*Step length (cm) was the only significantly different spatiotemporal gait variable between walking barefoot and walking in footwear (mean difference (MD) = -2.85cm, 95%CI= 0.31 to 5.39, p=0.03).*

**- On line 245, information is provided about weeks of walking experience. In the method section, the authors should provide details about how they acquired these data (e.g., Were parents asked to report this during an interview? If so, what definition was provided to the parents about what walking onset means?).**

*Response*

We have now added more detail regarding the instructions we provided parents on how to describe independent walking at Line 131-132:

*This included parent reported age, sex, height, weight and weeks since independent walking (defined as ongoing walking greater than 10 steps without parent hand holding for support).*

**- Standard deviations for the spatiotemporal parameters appear to be larger for toddlers when they walked wearing shoes. Is that variability significantly different from when toddlers walked barefoot? Variability is also a very important factor to examine when studying newly walking toddlers.**
*Response*

Thank you for this suggestion. We have now compared the standard deviations between the shod conditions for all variables and reported the p value of these comparisons in Tables 1 and 2. We have also discussed the findings in lines 290-291 and 301-303 of the results. We have also commented on the variables and their unknown clinical significance within the discussion at lines 326-327. During this time, we also took the opportunity to ensure there was consistency in decimal points between the tables as there were some small differences in rounding from the previous submission.

**- Lines 325 – 339: Although mean differences in spatiotemporal parameters were not different, I still question whether variability differed between barefoot and shod walking (i.e., differences in standard deviations).**

*Response*

As per previous request we have provided these in the table and further information in results.

Kind regards,

Cylie Williams, on behalf of the author team.
